# IMPLEMENTATION AND RESULTS: THE ZESTFUL LIVING PROGRAM FOR OLDER ADULTS RESIDING IN CNY ASSISTED LIVING FACILITIES

**DOI:** 10.1093/geroni/igad104.2068

**Published:** 2023-12-21

**Authors:** Minjung Seo, Mary Theresa Pagan

**Affiliations:** SUNY Oswego, Oswego, New York, United States; SUNY Oswego, Oswego, New York, United States

## Abstract

Studies indicate that older adults experiencing more interactions with younger ones through health promotion programs perceive better health and life satisfaction than their counterparts. Positive attitudes toward aging and self-efficacy working with older adults are essential attributes for professionals entering gerontology fields. The aim of this study is two-fold, to examine the changes in attitude toward aging and self-efficacy to work with older adults among young adults and to investigate changes in perceived physical and mental health among older adults participating in the Zestful Living Health Promotion Program in an assisted living facility in Upstate New York. A mixed method design (questionnaire and interview) was used on seventy-five older adults (Male: 15, Female: 49) and eighty-two college students. The five-week program theoretical framework utilized tenets from social learning theory and the behavioral change model. This evidenced-based program included exercises, memory training, stress reduction activities, and interactive games using implementation strategies such as health communication, health information, behavioral modification, and positive reinforcement. Results indicate attitudes toward aging and self-efficacy to working with older adults significantly differed before and after program implementation (P <.004, P <.0001). The potential benefits of this approach include improved perceived mental and physical health among older adults during five weeks with low resource allocation as a university supported. Sustainable programs like the Zestful Living Program provide research and outreach opportunities within assisted living facilities.

